# Immune cell-specific gene expression and its causal role in osteoporosis and bone mineral density: Insights from single-cell eQTL and GWAS data integration

**DOI:** 10.1097/MD.0000000000045869

**Published:** 2025-11-14

**Authors:** Xiaoming Wang, Xiao Xiao, Jiao Situ, Qinguang Xu, Jieji Zhang, Wenjie Xu, Denghui You, Yong Ju, Yi Zhou, Jining Jiang, Shirong Yang

**Affiliations:** a Fenghua Hospital of Traditional Chinese Medicine, Ningbo City, Zhejiang, China; b GuangYuan Hospital of Traditional Chinese Medicine, Guangyuan City, Sichuan, China; c Huzhou Central Hospital, Fifth School of Clinical Medicine Zhejiang Chinese Medical University Central Hospital, Huzhou City, Zhejiang, China; d Xiwu Branch of Fenghua District Hospital of Traditional Chinese Medicine Medical Community, Ningbo City, Zhejiang, China.

**Keywords:** bone mineral density, eQTL, immune cells, osteoporosis, single-cell RNA sequencing

## Abstract

Osteoporosis (OP) is a common metabolic bone disease, with genetic and immune system factors playing crucial roles in its pathogenesis. With the advancement of single-cell RNA sequencing (scRNA-seq), gene expression regulation at the immune cell subtype level has been more deeply explored. In this study, we integrated single-cell expression quantitative trait loci data with genome-wide association study data to systematically investigate the causal relationships between immune cell-specific gene expression and OP risk/ bone mineral density (BMD). Through summary-data-based Mendelian randomization, two-sample Mendelian randomization, Steiger directionality tests, and colocalization analysis, we identified 7 genes in specific immune cell types that are associated with OP/BMD phenotypes, including GLTPD1, NPRL3, NCR3, HBQ1, POU5F1, CDC42, and C10orf32. Specifically, GLTPD1, NPRL3, NCR3, HBQ1, and POU5F1 showed significant causal effects on OP risk, CDC42 was associated with total-body BMD in the 0 to 15 age group, and C10orf32 showed significant causal effects with total-body BMD in the >60 age group. Our findings provide new insights into the role of the immune system in bone metabolism and offer important theoretical support for further research on immune-mediated treatment strategies for OP.

## 1. Introduction

Osteoporosis (OP) is a systemic skeletal disorder characterized by reduced bone mass and the deterioration of bone microarchitecture, leading to decreased bone strength and a significantly increased risk of fractures.^[1,2]^ In the United States alone, approximately 2 to 3 million osteoporotic fractures occur annually, severely compromising patients’ quality of life and markedly increasing both morbidity and mortality rates.^[3,4]^

Under physiological conditions, bone tissue maintains a finely tuned remodeling process, initiated by osteoclast-mediated bone resorption,^[5]^ followed by the formation and mineralization of new bone by osteoblasts. However, this dynamic equilibrium is susceptible to disruption by various factors, including estrogen deficiency, aging, chronic diseases, and certain medications. When the rate of bone resorption surpasses that of bone formation, bone loss ensues, ultimately leading to the development of OP.^[6]^

While OP has traditionally been viewed as a metabolic disorder resulting from endocrine dysregulation,^[7]^ increasing evidence has highlighted extensive crosstalk between the skeletal and immune systems. To underscore the pivotal role of the immune system in this process, Srivastava et al introduced the concept of immunoporosis, referring to a subtype of OP predominantly driven by immune-mediated mechanisms.^[8,9]^ Nevertheless, studies exploring the potential causal relationships between gene expression in specific immune cell subtypes and OP risk remain limited, especially those leveraging genetic regulation to systematically identify immune-mediated therapeutic targets.

With the rapid advancement of single-cell RNA sequencing technology, researchers can now explore transcriptomic profiles at an unprecedented resolution.^[10]^ Recent efforts have integrated genetic variation with single-cell transcriptomic data to construct comprehensive cell-type-specific expression quantitative trait loci (eQTLs) maps across various immune cell populations.^[11]^ These loci have proven to be powerful tools for identifying disease-associated causal genes and elucidating disease mechanisms, showing significant value in both immune-related^[11,12]^ and metabolic disorders.^[13]^ However, studies that systematically apply Mendelian randomization (MR) to single-cell immune eQTLs data to evaluate the causal relationships between immune gene expression and OP risk are still scarce, and the underlying mechanisms remain insufficiently explored.

Therefore, this study uses immune single-cell expression quantitative trait loci (sc-eQTLs) and genome-wide association study (GWAS) data, employing summary-data-based Mendelian randomization (SMR), two-sample MR, and genetic colocalization analysis to systematically investigate the potential causal effects of gene expression in 14 distinct immune cell types on OP and bone mineral density (BMD) risks. Our goal is to identify key immune-related causal genes, provide new insights into the interplay between the immune system and OP, and lay a theoretical foundation for the application of personalized immunological intervention strategies in the treatment of this disease.

## 2. Materials and methods

### 2.1. Study design and data resources

**Figure 1.**
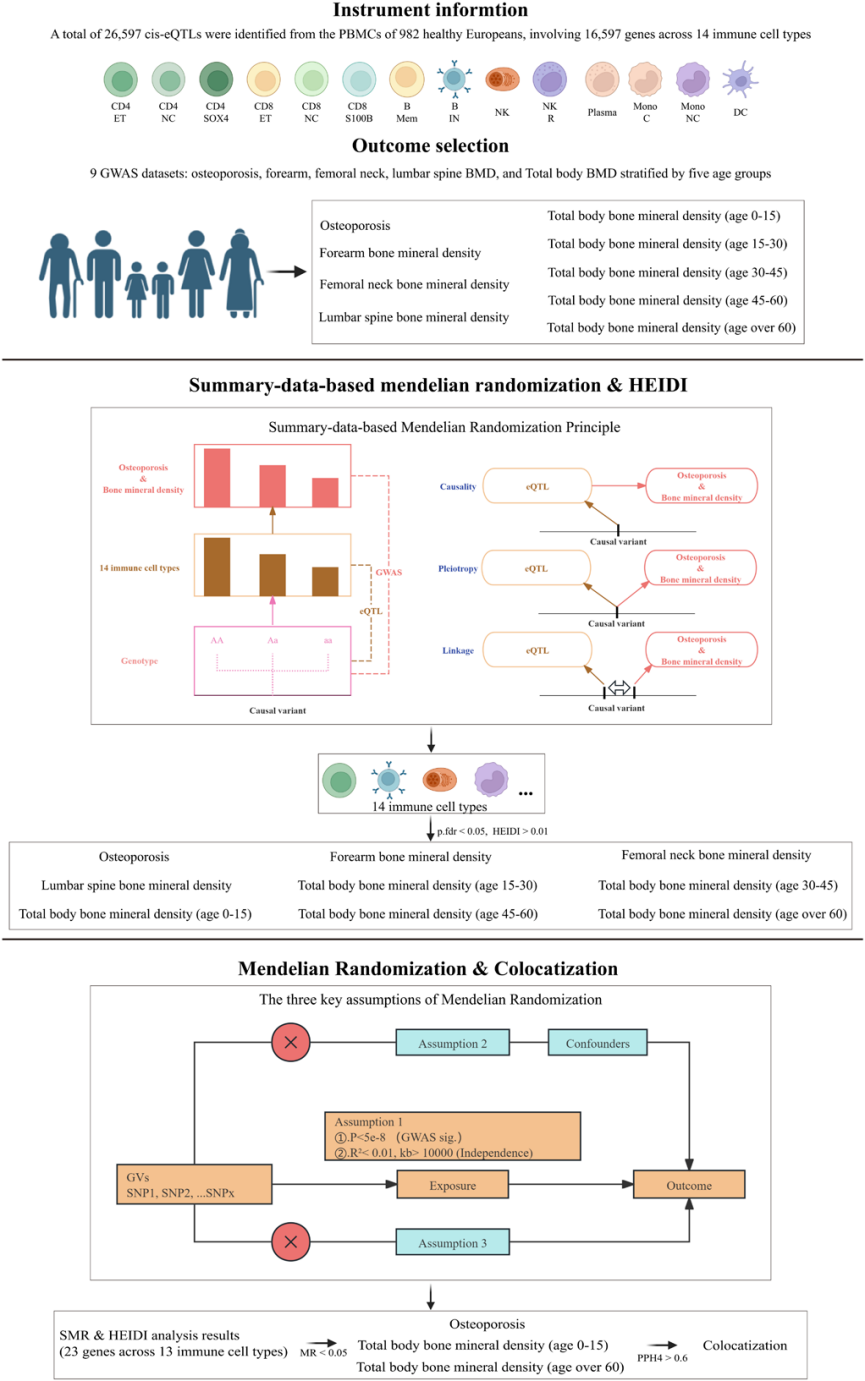
Overview of the study design.

This study is an observational two-sample, SMR analysis. The overall workflow of this study is shown in Figure 1. First, we extracted eQTLs data specific to 14 immune cell types from the OneK1K cohort, which is based on high-throughput single-cell RNA sequencing (scRNA-seq) data of peripheral blood mononuclear cells from 982 individuals, comprising approximately 1.27 million cells.^[11]^ This eQTL dataset was used as the exposure variable in our analysis.

The outcome variables were derived from 9 publicly available GWAS datasets, including the OP case–control GWAS from the FinnGen consortium (10,461 cases and 462,803 controls, data accessed on March 20, 2025).^[14]^ To comprehensively assess BMD changes in common skeletal sites, we incorporated quantitative-trait GWAS data for forearm (FABMD), femoral neck (FNBMD), and lumbar spine (LSBMD) from the GEFOS consortium, with sample sizes of 8143, 32,735, and 28,498 individuals, respectively, all measured by dual-energy X-ray absorptiometry (DXA) (data accessed on March 20, 2025).^[15]^ Additionally, to study the relationship between immune cell types and BMD across different age groups, we utilized age-stratified total body BMD (TBBMD) data from the IEU GWAS database. This dataset includes 5 age groups: 0 to 15 years (11,807 individuals), 15 to 30 years (4180 individuals), 30 to 45 years (10,062 individuals), 45 to 60 years (18,805 individuals), and 60+ years (22,504 individuals), all accessed on March 20, 2025. Except for the age-stratified data, all other samples were from individuals of European descent, with 86% of the age-stratified data coming from Europeans and 14% from mixed populations. All datasets used in this study are detailed in Table S1 (Supplemental Digital Content, https://links.lww.com/MD/Q636).

### 2.2. SMR analysis and HEIDI test

SMR is a statistical method that integrates quantitative trait loci (QTLs) and GWAS data to uncover potential associations between genetic variants and diseases or phenotypes. The heterogeneity in dependent instruments (HEIDI) test further assesses HEIDI to exclude false positives that may arise from genetic variation. In this study, we applied SMR to systematically evaluate potential associations between gene expression in 14 immune cell types and OP/BMD-related phenotypes, providing an initial screening step for subsequent causal inference. All analyses were conducted using the SMR software (version 1.3.1; Yang Lab, Westlake University, Hangzhou, Zhejiang, China).^[16,17]^

Instrumental variables (IVs) were selected based on the following criteria: we included independent cis-eQTLs significantly associated with target gene expression (*P* < 5 × 10^−8^), located within ±500 kb of each target gene.^[18]^ LD-based clumping (*R*^2^ < 0.01) was used to ensure the independence of the selected IVs, avoiding potential linkage disequilibrium (LD) effects. All analyses used the European reference panel from the 1000 Genomes Project for LD clustering and selection.^[19]^ In SMR analysis, for each significant association (*P* < .05), a HEIDI test was performed to exclude associations with *P* < .01 in HEIDI. Associations that passed the HEIDI test (*P* > .01) and remained statistically significant (*P* < .05) were considered to reflect genuine genetic effects, with shared pathogenic variation likely driving the association.

### 2.3. Two-sample Mendelian randomization

To verify the causal relationships between the candidate genes identified through SMR and HEIDI analysis and OP/BMD-related phenotypes, we performed two-sample MR analysis. MR is based on 3 fundamental assumptions: Relevance assumption – the IVs must be strongly associated with the exposure of interest; Independence assumption – the IVs should not be associated with potential confounders; and Exclusion restriction assumption – the IVs should affect the outcome solely through the exposure, not via alternative pathways.^[20]^

We first extracted independent cis-eQTLs significantly associated with each target gene (*P* < 5 × 10^−8^) and located within ±100 kb of the target gene to serve as IVs. LD-based clumping (*R*^2^ < 0.01) ensured IV independence, using the European panel from the 1000 Genomes Project for reference. We calculated the *F*-statistic (*F* = (β/SE)^2^) for each single nucleotide polymorphism (SNP)^[21]^ to assess IV strength and applied the Steiger filtering method to exclude SNPs whose variation in the outcome explained more than in the exposure, thereby reducing the risk of reverse causality bias.^[22]^

For genes with a single valid IV, causal estimates were calculated using the Wald ratio method^[23]^; For genes with multiple IVs, we used the inverse-variance weighted method, which adjusts for heterogeneity and measurement error to improve estimation accuracy. To avoid bias, we did not use proxy SNPs to replace missing variants. All MR analyses were conducted using the TwoSampleMR package in R (version 4.2.2; MRC Integrative Epidemiology Unit [IEU], University of Bristol, Bristol, England, United Kingdom), with multiple testing correction applied using the FDR threshold of <0.05. The entire analytical protocol followed the STROBE-MR reporting guidelines^[24,25]^ to ensure scientific rigor and reproducibility.

### 2.4. Colocalization analysis

To further validate the biological plausibility of the MR findings, we performed colocalization analysis for candidate genes demonstrating significant causal associations across different cohorts. This analysis was conducted using the “coloc” R package^[26]^ and employed a Bayesian statistical framework to assess whether gene expression and OP or BMD phenotypes are likely driven by the same underlying genetic variant. Specifically, for each significant MR-associated signal, we examined a ±500 kb region centered on the lead eQTL SNP to calculate the posterior probability of colocalization with OP or BMD risk. The colocalization analysis evaluated 5 mutually exclusive hypotheses: H0: Neither trait is associated; H1/H2: Only one trait is associated; H3: Both traits are associated but driven by distinct causal variants; H4: Both traits are associated and share the same causal variant.^[27]^ The prior probabilities were set as follows: P1 = 1 × 10^−4^ (association with gene expression only), P2 = 1 × 10^−4^ (association with the disease phenotype only), and P12 = 1 × 10^−5^ (association with both traits). A posterior probability for H4 (PP.H4) ≥ 0.6 was considered strong evidence for colocalization, suggesting that gene expression and OP/BMD risk are likely driven by a shared genetic variant, thereby strengthening the credibility of the inferred causal relationship.

### 2.5. Ethics

All original studies were conducted with informed consent from participants and received approval from the appropriate institutional ethics committees. The present study involves only secondary analysis of publicly available data and does not require additional ethical review.

## 3. Results

### 3.1. SMR and HEIDI analysis

We extracted a total of 26,597 independent cis-eQTLs from the single-cell transcriptome data of 982 individuals in the OneK1K cohort, involving 16,597 genes across 14 immune cell types, which were used as candidate instrumental variables. Through initial SMR and HEIDI analyses, we identified 58 genetic signals significantly associated with OP and various BMD phenotypes (FDR < 0.05) (Table S2, Supplemental Digital Content, https://links.lww.com/MD/Q636). Specifically, there were 46 signals related to OP risk, 1 related to FNBMD, 1 related to LSBMD, 7 related to TBBMD in the 0 to 15 age group, and 3 related to TBBMD in the >60 age group, all of which were statistically significant.

**Figure 2.**
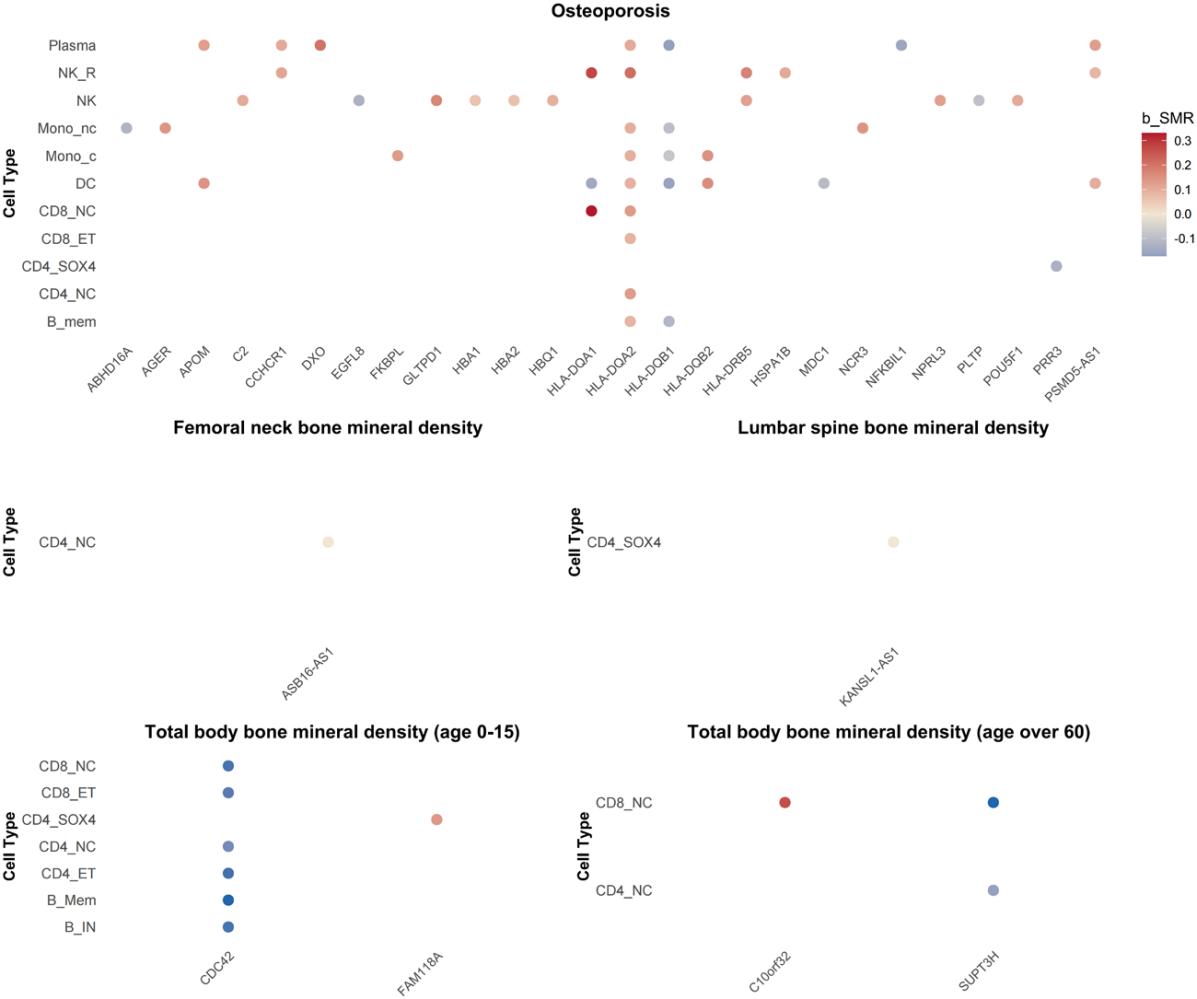
Visualization of summary-data-based Mendelian randomization and heterogeneity in dependent instruments analysis results for osteoporosis and bone mineral density in immune cells.

Further analysis revealed that among the 46 genetic signals associated with OP risk, 11 immune cell types were involved, with 12 signals showing a negative association with OP risk and 34 signals showing a positive association (Fig. 2). These signals were associated with 26 genes, with 8 genes showing consistent significance across at least 2 immune cell types, including HLA-DQA2 (9 cell types), HLA-DQB1 (5 cell types), HLA-DQA1 and PSMD5-AS1 (both in 3 cell types), and HLA-DQB2, HLA-DRB5, CCHCR1, and APOM (all in 2 cell types). Additionally, several key immune cell-specific genes associated with BMD phenotypes were identified: ASB16-AS1 in CD4_NC cells showed a significant negative association with femoral neck BMD risk, KANSL1-AS1 in CD4_SOX4 cells was significantly negatively associated with lumbar spine BMD risk, CDC42 in 6 immune cell types was significantly associated with BMD risk in the 0 to 15 age group (TBBMD, age 0–15), with consistent effect directions, and FAM118A in CD4_SOX4 cells was significantly positively associated with this phenotype risk. For the >60 age group, SUPT3H in CD4_NC and CD8_NC cells was significantly negatively associated with TBBMD, while C10orf32 in CD8_NC cells showed a significant positive association.

### 3.2. Two-sample Mendelian randomization results

**Figure 3.**
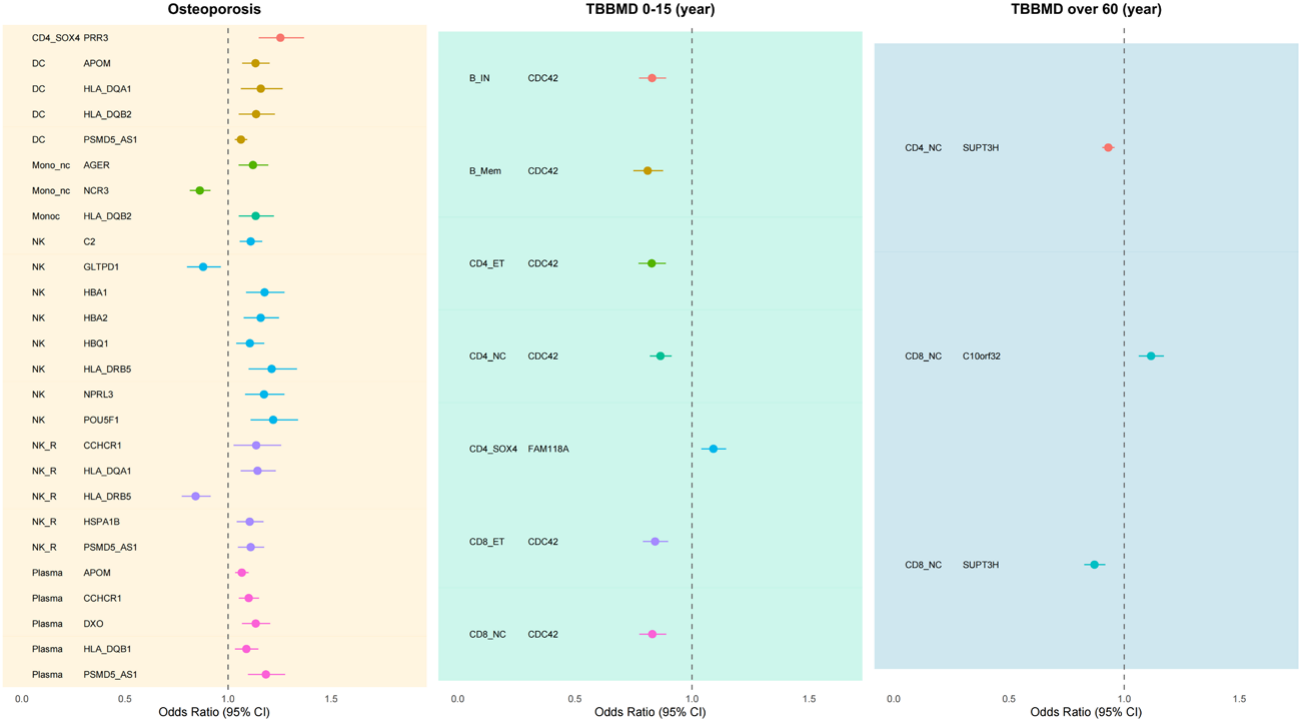
Two-sample Mendelian randomization analysis of immune cell gene expression and its association with osteoporosis/bone mineral density: forest plot.

To further verify the causal relationships between the candidate genes identified through SMR and HEIDI analyses and OP/BMD-related phenotypes, we performed two-sample MR analysis. A total of 27 genetic signals significantly associated with OP risk were identified, involving 19 genes from 7 immune cell types. Additionally, 7 signals related to BMD risk in the 0 to 15 age group (TBBMD, age 0–15) were identified, involving 2 genes (CDC42 and FAM118A) from 7 immune cell types, and 3 signals related to BMD risk in the >60 age group (TBBMD, age > 60), involving 2 genes (SUPT3H and C10orf32) from 2 immune cell types (Table S4, Supplemental Digital Content, https://links.lww.com/MD/Q636). In the OP phenotype, most genes exhibited consistent effect directions in the same immune cell types. For example, GLTPD1, HBA1, HBA2, and HBQ1 all showed a significant positive association with OP risk in natural killer (NK) cells (odds ratio [OR] 95% confidence interval [CI] = 1.18 [1.09–1.27], 1.06 [1.03–1.09], 1.06 [1.03–1.09], 1.10 [1.05–1.15], respectively). Some genes also displayed consistent effect directions across multiple immune cell types, such as PSMD5-AS1, which showed positive associations in DC, NK_R, and plasma cells (OR 95% CI = 1.10 [1.03–1.17], 1.08 [1.03–1.14], 1.13 [1.05–1.22], respectively); APOM in DC and plasma cells (OR 95% CI = 1.21 [1.10–1.33], 1.13 [1.05–1.22]); CCHCR1 in NK_R and plasma cells (OR 95% CI = 1.14 [1.06–1.23], 1.12 [1.05–1.19]). Additionally, some genes were significantly associated with OP risk only in specific immune cell types, such as HLA-DQB1 in plasma cells (negative association, OR 95% CI = 0.84 [0.77–0.91]), PRR3 in CD4_SOX4 cells (negative association, OR 95% CI = 0.87 [0.80–0.96]), and AGER in Mono_nc cells (positive association, OR 95% CI = 1.17 [1.08–1.27]) (Fig. 3).

In BMD phenotype analysis, CDC42 in CD8_NC, CD8_ET, CD4_NC, CD4_ET, B_Mem, and B_IN cells was significantly negatively associated with TBBMD in the 0 to 15 age group, with consistent effect directions, indicating stable causal effects in childhood BMD regulation. FAM118A was specifically expressed in CD4_SOX4 cells and was significantly positively associated with BMD at this stage. For the >60 age group, SUPT3H in CD4_NC and CD8_NC cells showed a significant negative association with TBBMD, while C10orf32 in CD8_NC cells showed a significant positive association. These results suggest that expression of candidate genes in specific immune cells may play causal roles in BMD changes at different ages, providing new genetic evidence for understanding immune regulation of bone metabolism.

### 3.3. Causal directionality testing

We conducted Steiger directionality tests for all candidate signals to assess whether each instrumental variable explained more variance in the exposure (gene expression) than in the outcome (OP). All instruments passed the test, supporting a biologically plausible causal direction, and the *F*-statistics for all instruments were >10, ensuring the robustness of the results (Table S3, Supplemental Digital Content, https://links.lww.com/MD/Q636).

### 3.4. Colocalization analysis

**Figure 4.**
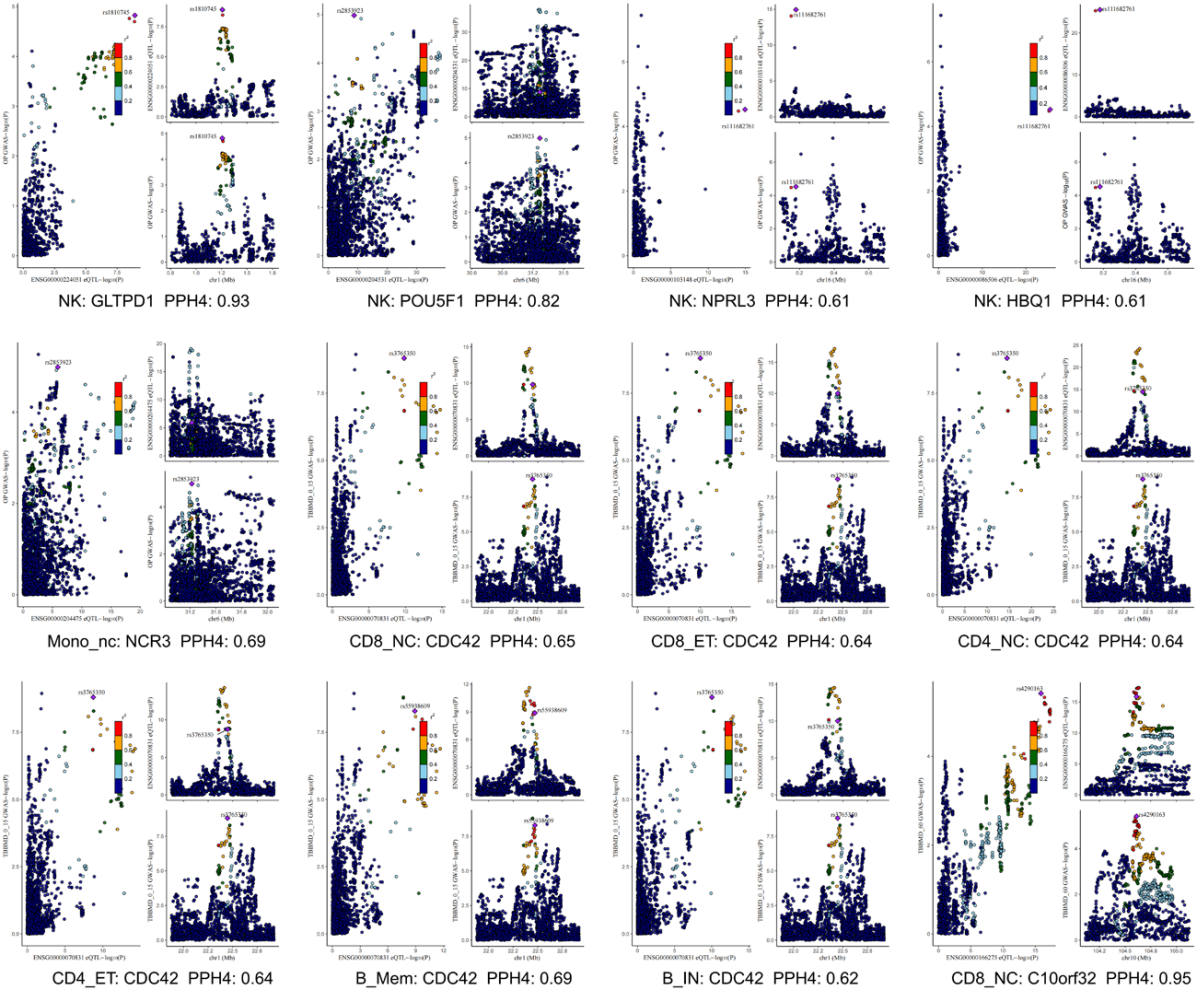
Colocalization analysis of immune cell gene expression and osteoporosis/bone mineral density (PPH4 > 0.6).

To further validate the genetic overlap between MR findings and OP/BMD phenotypes, we conducted Bayesian colocalization analysis (Fig. 4). The results showed that 5 genes associated with OP risk displayed strong colocalization evidence in 2 immune cell types: GLTPD1 (PP.H4 = 0.93), POU5F1 (PP.H4 = 0.82), NPRL3 (PP.H4 = 0.61), HBQ1 (PP.H4 = 0.61) from NK cells, and NCR3 (PP.H4 = 0.69) from Mono_nc cells. Notably, GLTPD1 showed the strongest colocalization signal and had a significant causal association with OP (*P* = 1.5 × 10^−5^, OR = 1.18, 95% CI: 1.09–1.27), further supporting its potential role as a pathogenic factor.

Additionally, for signals associated with TBBMD in the 0 to 15 age group, CDC42 showed stable negative associations across 6 immune cell types (*P* = 1.39 × 10^−7^, OR = 0.81–0.86), with high posterior probabilities of colocalization (PP.H4 = 0.62–0.69) in all cell types, suggesting that this gene may be driven by the same causal variant in childhood BMD regulation. Meanwhile, C10orf32 in CD8_NC cells showed a significant positive association with TBBMD in the >60 age group (*P* = 1.01 × 10^−5^, OR = 1.11, 95% CI: 1.06–1.17), with a posterior probability of 0.95, providing strong colocalization evidence and supporting its role in regulating bone metabolism in older adults (Table 1; Table S5, Supplemental Digital Content, https://links.lww.com/MD/Q636).

**Table 1:** List of potential causal genes identified by integrating MR results and colocalization analysis.

eQTL_profiles	Exposure	Disease	*P*-value	OR (95% CI)	PPH4
NK	GLTPD1	OP	1.5 × 10^−5^	1.18 (1.09–1.27)	0.93
NK	POU5F1	OP	3.69 × 10^−5^	1.10 (1.05–1.16)	0.82
NK	NPRL3	OP	2.99 × 10^−5^	1.13 (1.06–1.20)	0.61
NK	HBQ1	OP	2.99 × 10^−5^	1.10 (1.05–1.15)	0.61
Mono_nc	NCR3	OP	.01	1.13 (1.02–1.25)	0.69
CD8_NC	CDC42	TBBMD (0-15)	1.39 × 10^−7^	0.83 (0.77–0.88)	0.65
CD8_ET	CDC42	TBBMD (0-15)	1.39 × 10^−7^	0.84 (0.78–0.89)	0.64
CD4_NC	CDC42	TBBMD (0-15)	1.39 × 10^−7^	0.86 (0.81–0.91)	0.64
CD4_ET	CDC42	TBBMD (0-15)	1.39 × 10^−7^	0.82 (0.77–0.88)	0.64
B_Mem	CDC42	TBBMD (0-15)	1.39 × 10^−7^	0.81 (0.74–0.87)	0.69
B_IN	CDC42	TBBMD (0-15)	1.39 × 10^−7^	0.82 (0.77–0.88)	0.62
CD8_NC	C10orf32	TBBMD (over 60)	1.01 × 10^−5^	1.11 (1.06–1.17)	0.95

CI = confidence interval, eQTL = expression quantitative trait locus, MR = Mendelian randomization, NK = natural killer, OP = osteoporosis, OR = odds ratio.

## 4. Discussion

This study systematically assessed the relationship between immune cell-specific gene expression and OP as well as BMD phenotypes, integrating sc-eQTL SMR analysis, MR analysis, and Bayesian colocalization analysis. Through SMR analysis, we identified 58 genetic signals significantly associated with OP and different BMD phenotypes, including 46 signals related to OP risk, 1 related to FNBMD, 1 related to LSBMD, 7 related to TBBMD in the 0 to 15 age group, and 3 related to TBBMD in the >60 age group (FDR < 0.05). Further MR analysis revealed 36 key genetic signals related to OP and BMD, involving 23 genes, suggesting a potential regulatory role of immune cell-specific gene expression in bone metabolism. Bayesian colocalization analysis further identified 7 genes with colocalized expression in specific cell types, including GLTPD1, NPRL3, NCR3, POU5F1, and HBQ1 in NK cells and non-classical monocytes (Mono_nc) with OP; CDC42 in 6 immune cell types with TBBMD in the 0 to 15 age group; and C10orf32 in CD8_NC immune cells with TBBMD in the >60 age group. Overall, these results provide strong genetic evidence for the potential mechanisms linking immune cell-specific gene expression with OP and BMD-related phenotypes.

The osteoimmunological model highlights the interaction between the immune and skeletal systems, with both systems sharing multiple overlapping transcription factors, signaling molecules, cytokines, and chemokines.^[28]^ It was discovered that osteoclasts were among the first immune-functional cells identified in the skeletal system, laying the foundation for the field of bone immunology.^[28]^ Recent studies have strengthened the pathophysiological framework of bone immunology in OP. For example, Zhao et al (2016) demonstrated that postmenopausal women with OP exhibit increased levels of pro-inflammatory cytokines, such as TNF, IL-1, IL-6, and IL-17,^[29]^ supporting the relationship between bone immunology and OP. In this study, Bayesian colocalization analysis identified 7 genes with colocalized expression in 14 immune cell types and relevant phenotypes. These genes likely influence bone metabolism through specific biological processes: First, studies have consistently shown that high plasma S1P levels are associated with increased fracture incidence and OP prevalence,^[30–33]^ and that GLTPD1 deficiency significantly reduces S1P levels.^[34]^ Our results indicate that higher levels of GLTPD1 are associated with an increased risk of OP. Therefore, we further hypothesize that GLTPD1 may enhance OP risk by elevating S1P levels. Second, NPRL3 is a core component of the GATOR1 complex, which negatively regulates the mTORC1 signaling pathway by inhibiting Rag GTPases.^[35,36]^ During aging, mTORC1 activation promotes increased expression of sodium channels (Scn1a), alters cell membrane potential, and induces depolarization of pre-osteoblasts, accelerating their aging. Our results indicate that high NPRL3 expression may increase OP risk by upregulating mTORC1 signaling, accelerating pre-osteoblast depolarization and aging, and impairing bone formation capacity. Additionally, recent studies have revealed the mechanism of BAG6-NCR3 interaction with Th17 cells in the bone. This interaction upregulates CD6 expression in Th17 cells, leading to the production of SPP1 (osteopontin), which in turn enhances osteoclast activity and promotes bone loss.^[37,38]^ This further supports the positive association between NCR3 expression and OP. Meanwhile, CDC42, a key molecule in the RANKL signaling pathway, is known to regulate osteoclastogenesis and subsequently modulate BMD.^[39–41]^ Our findings indicate that CDC42 negatively regulates whole-body BMD in the 0 to 15 age group, possibly by enhancing osteoclast activity, leading to reduced bone density.

Moreover, our Bayesian colocalization analysis revealed stable genetic colocalization evidence between POU5F1, HBQ1, and C10orf32 and relevant phenotypes in specific immune cell types. Although these findings have not been reported in previous studies in relation to the relevant phenotypes, our results provide additional biological insights. Notably, POU5F1/OCT4 is a critical transcription factor for maintaining stem cell pluripotency, with well-established roles in mesenchymal stem cells.^[42,43]^ However, the specific relationship between OCT4 and osteoclasts remains unclear. Additionally, HBQ1, a component of hemoglobin, may be involved in oxidative stress or inflammation regulation,^[44,45]^ while C10orf32 has mainly been studied in the context of malnutrition and motor dysfunction caused by its deficiency.^[46,47]^ Recent studies have linked high expression of C10orf32 with muscle wasting,^[48]^ but its role in bone metabolism warrants further investigation. These genetic associations provide new clues for understanding the complexity of bone immune regulation networks.

Despite the strengths of this study, there are several limitations. First, the gene expression data used were derived only from resting peripheral immune cells, which do not reflect transcriptional dynamics in bone-specific microenvironments or under stimulation conditions such as inflammatory cytokines or mechanical stress. Second, the sc-eQTL data were sourced exclusively from European populations, and the generalizability of our findings to other populations requires further validation. Lastly, while we applied rigorous filtering and colocalization analyses to strengthen the robustness of causal inference, our conclusions are still based on statistical associations and lack direct experimental validation. Therefore, future studies should focus on functional experiments in cellular and animal models to further elucidate the specific mechanisms and pathways through which these genes influence bone metabolism.

## 5. Conclusions

In conclusion, this study systematically identified stable associations between GLTPD1, NPRL3, NCR3, HBQ1, POU5F1, CDC42, and C10orf32, and OP as well as BMD phenotypes in specific immune cell types, by integrating various genetic analysis methods. For certain genes (GLTPD1, NPRL3, NCR3, and CDC42), we successfully constructed plausible biological mechanisms based on existing knowledge, providing a solid theoretical foundation for understanding their roles. For POU5F1, HBQ1, and C10orf32, although their specific bone regulatory pathways remain unclear, strong colocalization evidence suggests that they may act as key factors in bone immune regulation, offering new directions for future research. These findings not only enhance our understanding of the immune system’s role in bone metabolism but also provide important genetic support for developing OP prevention and treatment strategies targeting immune cells.

## Acknowledgments

The authors sincerely thank the FinnGen consortium, the OneK1K project, the GEFOS consortium, and the IEU GWAS database for providing high-quality GWAS and single-cell transcriptomic data, which made this study possible. The authors also extend their gratitude to all participants and researchers who contributed valuable data to this study.

## Author contributions

**Conceptualization:** Xiaoming Wang.

**Data curation:** Xiao Xiao.

**Funding acquisition:** Shirong Yang.

**Investigation:** Jiao Situ.

**Methodology:** Qinguang Xu.

**Resources:** Jieji Zhang, Wenjie Xu.

**Software:** Denghui You, Yong Ju.

**Validation:** Xiaoming Wang, Yi Zhou.

**Visualization:** Jining Jiang.

**Writing – original draft:** Xiaoming Wang, Shirong Yang.

**Writing – review & editing:** Shirong Yang.

## Supplementary Material


